# Direct Versus Indirect Submaximal VO_2_max Assessment in Masters Basketball Players

**DOI:** 10.3390/jfmk10040431

**Published:** 2025-11-05

**Authors:** Kristine Dakule, Una Veseta, Voldemars Arnis, Ketija Grinberga, Oskars Kalejs, Signe Tomsone

**Affiliations:** 1 Master’s Programme in Sports Science, Faculty of Medicine and Health Sciences, University of Latvia, 1 Jelgavas Street, LV-1004 Riga, Latvia; 2 Department of Individual and Team Sports, Rīga Stradiņš University, 16 Dzirciema Street, LV-1007 Riga, Latvia; 3 Department of Rehabilitation, Faculty of Health and Sport Sciences, Rīga Stradiņš University, 16 Dzirciema Street, LV-1007 Riga, Latviasigne.tomsone@rsu.lv (S.T.); 4 Institute of Public Health, Faculty of Health and Sport Sciences, Rīga Stradiņš University, 16 Dzirciema Street, LV-1007 Riga, Latvia; 5 The Red Cross Medical College of Rīga Stradiņš University, Jāņa Asara Street 5, LV-1009 Riga, Latvia; 6 Department of Internal Diseases, Faculty of Medicine, Rīga Stradiņš University, 16 Dzirciema Street, LV-1007 Riga, Latvia

**Keywords:** VO_2_max, masters athletes, basketball, aerobic capacity, indirect fitness testing, cardiopulmonary exercise test, CPET

## Abstract

**Background:** Accurate assessment of aerobic capacity is essential for performance monitoring in masters athletes, particularly in high-intensity team sports. The objective of this study was to evaluate the validity and agreement of three indirect maximal oxygen uptake (VO_2_max) protocols (Åstrand–Ryhming, YMCA, and Polar OwnIndex Fitness test) against the gold-standard cardiopulmonary exercise testing (CPET) in masters basketball players. **Methods:** A cross-sectional comparative study included 50 male masters basketball players (aged 51–81 years, M = 64.3 ± 7.9). Validity was determined by comparing results from the three indirect protocols to direct VO_2_max measurement via CPET. Agreement was assessed using Pearson correlations (r), systematic error, mean absolute error (MAE), and Bland–Altman limits of agreement. **Results:** The Åstrand–Ryhming test and YMCA tests showed the closest agreement with CPET (systematic error < 4%, MAE ≈ 17–18%, *r* > 0.50). The Polar OwnIndex test substantially overestimated VO_2_max (mean error ≈ 30%, MAE = 32%). The Åstrand–Ryhming test at low workload yielded the strongest correlation (r = 0.75). **Conclusions:** The Åstrand–Ryhming and YMCA submaximal tests demonstrated acceptable validity and low systematic bias for estimating VO_2_max in masters basketball players, positioning them as practical alternatives to CPET. Conversely, the Polar OwnIndex test showed poor agreement and clinically significant overestimation. These findings support the use of submaximal cycling protocols for fitness monitoring and tailored training prescription in this specific older athlete population. Future longitudinal research is warranted to confirm their ability to track fitness changes over time in this population.

## 1. Introduction

Human aging is an inevitable biological process that progressively affects all physiological functions of the body. One of the most significant factors influencing health-related quality of life during this process is the regularity of physical activity [1]. The World Health Organization (WHO) highlights physical activity as a fundamental component of a healthy lifestyle. Regular physical activity enhances overall health status and helps prevent chronic diseases, including diabetes mellitus, cardiovascular diseases, and oncological conditions [2].

During exercise, the earliest adaptations are observed in the cardiovascular and respiratory systems [3]. These adaptations define cardiorespiratory fitness, which is one of the most important predictors of health outcomes and mortality risk. According to Lang et al. (2024), a meta-analysis of more than 20.9 million observations from 199 independent cohort studies found that individuals with higher cardiorespiratory fitness had a significantly reduced risk of all-cause mortality [4].

Aerobic capacity is especially critical in endurance and team sports. Basketball is a high-intensity sport characterized by frequent alternations between anaerobic and aerobic energy systems, particularly during the second half of the game when fatigue accumulates and oxygen delivery decreases. In this context, maximal oxygen uptake (VO_2_max) is a critical determinant not only for overall game endurance but also for the ability to recover rapidly between high-intensity bursts and to sustain repeated high-level efforts, which directly influences performance throughout the match [5,6]. Regular assessment of aerobic fitness is necessary for athletes to achieve peak physical condition. Given that time constraints are frequently reported as a major barrier to physical activity, shorter yet effective high-intensity interval training protocols that elicit comparable benefits to prolonged moderate-intensity exercise have become increasingly popular [7].

Physical fitness, particularly aerobic capacity, is a key determinant of high-level athletic performance. VO_2_max is an objective and internationally recognized measure of aerobic fitness, which can be assessed through both direct and indirect methods [8]. The “gold standard” for aerobic capacity assessment is the direct measurement of VO_2_max via cardiopulmonary exercise testing (CPET) with breath-by-breath gas analysis [9]. Beyond merely establishing VO_2_max, CPET offers a comprehensive assessment of integrated physiological responses (metabolic, cardiac, and pulmonary), which is crucial for establishing safety and detailed health status in older competitive athletes [10,11]. Although highly accurate, the widespread use of CPET is limited by its high cost, need for specialized equipment, and trained personnel. Consequently, simpler and more accessible indirect VO_2_max estimation protocols such as the Åstrand–Ryhming cycle ergometer test, YMCA submaximal test, and Polar OwnIndex test are frequently employed in practice [12,13]. However, research indicates that these indirect methods may show significant discrepancies compared to direct measurements, impacting diagnostic validity and training prescription accuracy [14].

Many former athletes continue to engage in competitive and recreational training beyond 35–40 years of age, entering the masters athlete category. However, age-related physiological declines, including reductions in VO_2_max, primarily driven by a decrease in maximal heart rate, a reduction in stroke volume, and changes in peripheral oxygen extraction, necessitate specialized approaches to monitoring physical fitness in this population [15]. Masters athletes remain relatively underrepresented in VO_2_max validation studies [16]. This knowledge gap is particularly critical in intermittent sports like basketball, where the accurate assessment of VO_2_max is essential for optimally adjusting training loads, mitigating injury risk, and preserving performance as age-related decline progresses. Evidence suggests that aerobic capacity in basketball players varies substantially according to age, sex, and relative age effect [17], and aging influences cardiopulmonary adaptations even in elite athletes [18]. These findings underscore the need to adapt fitness assessment protocols for different age groups, including masters athletes. Based on these considerations, the following hypothesis was formulated: the Åstrand–Ryhming and YMCA tests will demonstrate better agreement and lower systematic bias compared to the Polar OwnIndex test when estimating VO_2_max in this population. This assumption is supported by prior research indicating that heart rate variability is greater at lower exercise intensities than at higher intensities [19]. Because the Polar OwnIndex test relies on resting-state parameters, it may be more susceptible to variability and errors, especially in older athletes whose autonomic regulation may differ from younger populations. Nonetheless, all indirect tests are expected to yield lower accuracy and greater individual variability than the direct CPET method. Therefore, the primary objective of this study was to determine the validity and agreement of the Åstrand–Ryhming, YMCA, and Polar OwnIndex test indirect VO_2_max assessment protocols compared to direct VO_2_max measurement via CPET in a population of masters basketball players. The results will provide a scientifically robust solution by offering practical, evidence-based recommendations on reliable and accessible VO_2_max tests specifically applicable to this masters athlete cohort, thereby enhancing fitness monitoring and training program optimization.

## 2. Materials and Methods

### 2.1. Study Design

This was a quantitative, comparative, cross-sectional validation study conducted at the Sports Healthcare Research Centre (SHRC), a scientific unit of Rīga Stradiņš University (RSU) and the Latvian Academy of Sport Education (LASE), from May to October 2024. The primary objective was to compare and validate VO_2_max values obtained using three indirect aerobic capacity assessment methods against the reference standard of direct CPET in masters basketball players.

### 2.2. Participants

**Figure 1:**
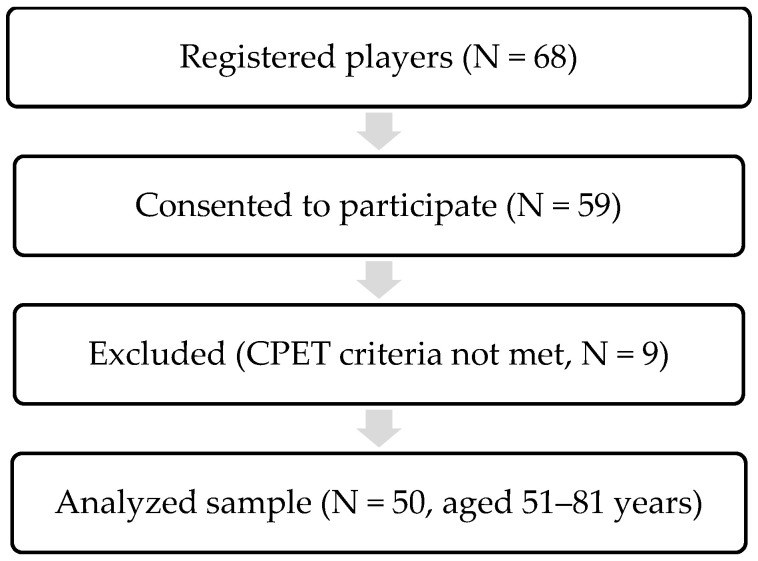
Participant recruitment and flow through the study. Note: CPET—cardiopulmonary exercise testing; N—number of participants.

Participants were recruited on a voluntary basis in collaboration with the Vecmeistari Basketball Club (http://maxibasket.lv/), which registers 68 players specifically in the 50+ age group. Of these, 59 male basketball players consented to participate. All participants regularly competed at the National Masters Basketball Championship level, playing an average of 18–24 games per season. Eligibility criteria: Participants were eligible if they were aged over 45 years, actively engaged in basketball training and competitions, provided written informed consent, and were able to complete the testing protocols. Exclusion criteria included diagnosed, untreated, or unstable cardiovascular diseases, active oncological or psychiatric conditions, neurodegenerative diseases (e.g., Parkinson’s or Alzheimer’s disease), or the presence of implanted electronic devices such as pacemakers. Nine participants were excluded due to failure to meet CPET criteria, including inability to reach the minimum heart rate of 120 bpm, medication use, or contraindications for testing. Thus, 86.8% of the initially screened athletes completed the study. Data from 50 participants (aged 51–81 years, M = 64.3 ± 7.9) were included in the analysis. The flow of participants through the study is presented in Figure 1.

The sample was designed to represent a specific population of physically active middle-aged and older men regularly engaged in basketball. Although no formal sample size calculation was performed prior to the study, the cohort was relatively homogeneous and demonstrated high compliance, which is critical for comparative physiological research. The study’s sample size (N = 50) was evaluated post hoc using a sensitivity power analysis (G*Power 3.1; [20]) to determine the statistical power for detecting different effect sizes, as recommended for exploratory research [21]. For the Pearson correlation test (two-tailed, α = 0.05, and power = 0.80), the minimum detectable correlation coefficient in the current sample was r ≥ 0.38 (equivalent to a medium-to-large effect size). This confirmed that the study had adequate power to detect moderate and strong associations. Given the small and well-defined target population (N = 68), inclusion of 59 participants represents near-complete recruitment, thereby enhancing the representativeness and internal validity of the study. While conventional power analyses would suggest larger sample sizes (e.g., N = 128 for medium effect detection at 80% power) in general populations, such calculations have limited applicability in niche populations where the entire accessible population is enrolled. In these contexts, representativeness and data quality often supersede sample size, especially in comparative studies. The participant selection utilized a convenience sampling approach, targeting the entire accessible population registered within the Vecmeistari Basketball Club; thus, no randomization was employed in the recruitment process.

### 2.3. Measurement Instruments

Direct measurement was performed using a RAMP protocol [22] on a Lode Excalibur Sport cycle ergometer (Lode BV, Zernikepark 16, 9747 AN Groningen, The Netherlands), designed for high-precision testing in clinical and sports science settings. The ergometer features an electromagnetic braking system and allows workload control between 8 and 2500 watts. The adjustable saddle and handlebar positions ensured optimal biomechanics and comfort for each participant. Cardiopulmonary data were collected using a Vyntus CPX metabolic cart (Vyaire Medical, 26,125 North Riverwoods Blvd, Mettawa, IL 60045, USA) with integrated high-accuracy gas analyzers and a digital flow sensor. The system measured oxygen uptake (VO_2_), carbon dioxide output (VCO_2_), minute ventilation, respiratory exchange ratio (RER), tidal volume, and breathing frequency in real time. Continuous 12-lead Electrocardiogram (ECG) monitoring was used to ensure participant safety throughout the test. Calibration of the CPX system was performed prior to each testing day using a two-point gas calibration and a 3-L syringe for flow verification, in accordance with the manufacturer’s guidelines.

Indirect assessments included the Polar OwnIndex Fitness Test (Polar Electro Oy, Professorintie 5, FI-90440 Kempele, Finland), the Åstrand–Ryhming test, and the YMCA submaximal cycling protocol. The Polar OwnIndex test was conducted in a supine position using a Polar V800 watch paired with a Polar H10 heart rate sensor. The resulting Polar OwnIndex test value, representing estimated VO_2_max, was derived from a proprietary algorithm that incorporates resting heart rate, age, sex, height, body weight, and self-reported physical activity level, and has been used in previous validation study [23]. The Åstrand–Ryhming test was performed on the Monark Ergomedic 839E cycle ergometer (Monark Exercise AB, Kroonsväg 1, 780 50 Vansbro, Sweden) using steady-state heart rate at a constant submaximal workload. The obtained values were processed through a validated online calculator (Health-calc.com), which is based on the Åstrand–Ryhming nomogram and includes sex-specific correction factors as described by Åstrand–Ryhming and Rodahl (1986) [24]. The YMCA protocol was executed using the Concept2 BikeErg (Concept2 Inc., 105 Industrial Park Drive, Morrisville, VT 05661, USA), an air-braked cycle ergometer with adjustable resistance and precise workload monitoring. Participants cycled through three incremental 3-min workload stages until submaximal exertion (~80–85% maximum heart rate (HRmax)). VO_2_max was estimated using a standard linear extrapolation model based on heart rate–workload data, with calculations performed via an online tool developed according to the algorithm described by Beekley et al. (2004) [25], which reflects established YMCA methodology [26]. All heart rate sensors were synchronized and verified before each session, and the same equipment was used for all participants. The use of standardized equipment, validated protocols, and consistent data processing procedures helped ensure methodological reliability. The indirect test results were later statistically compared to direct VO_2_max values to evaluate agreement, bias, and estimation accuracy.

### 2.4. Procedure

The study was conducted in accordance with the Declaration of Helsinki (1975, revised in 2013) and approved by the Ethics Committee of Rīga Stradiņš University (Protocol No. 2-PĒK-4/311/2024; approval date: 18 March 2024). All participants provided written informed consent prior to participation. Data confidentiality was maintained via anonymized identifiers and aggregated reporting. All tests were performed on the same day in a fixed order, (CPET, followed by Polar OwnIndex test, Åstrand–Ryhming, and YMCA). A standardized rest interval of 90 min was provided between CPET and the first indirect test, and 30 min between subsequent indirect tests, to minimize fatigue and ensure recovery. The fixed order was implemented to mitigate the potential influence of residual exercise-induced fatigue from the maximal CPET on the subsequent submaximal and resting tests. Direct CPET was conducted under standardized laboratory conditions (20–22 °C), preceded by resting ECG and echocardiography. CPET was administered individually by a certified sports physician–cardiologist, whereas indirect tests were conducted by doctoral-level sport science specialists. To reduce psychological influences such as anxiety, lack of motivation, or unfamiliarity with the testing procedures, participants were thoroughly briefed, given time to acclimate to the equipment, and verbally encouraged to perform maximally. Testing sessions were conducted in a supportive, standardized environment, ensuring that psychological and environmental factors did not confound physiological measurements.

### 2.5. Statistical Data Analysis

Data were compiled in an encrypted Excel file, recording participant IDs and all measurements. All estimated VO_2_max values from the indirect tests were calculated using established age- and sex-specific correction factors to ensure optimal comparison with the gold-standard CPET results. Statistical analyses were performed using Jamovi software for a desktop PC Jamovi free license version 2.3.28.0 (The Jamovi Project 2025) with the Bland–Altman Method Comparison module and Regression Correlation Matrix. Normality of data distribution was checked using the Shapiro–Wilk test. Mean VO_2_max values and standard deviations were calculated using Microsoft Excel (Microsoft Corporation, Excel (Microsoft 365 Subscription), Version 2024, Computer Software) functions (AVERAGE, STDEV.S). Agreement and differences between methods were analyzed using Bland–Altman plots and mean absolute error (MAE). Inter-test correlations were evaluated using Pearson’s correlation coefficient. Statistical significance was set at *p* < 0.05. Confidence intervals (95%) were calculated where appropriate to assess the precision of estimates. In addition to correlation coefficients, the strength of association between tests was interpreted based on conventional thresholds (e.g., *r* > 0.7 = strong). Bland–Altman plots were used to evaluate systematic bias and the 95% Limits of Agreement (LoA) between indirect and direct VO_2_max measures. The 95% Confidence Intervals (CI) for the LoA were also calculated to enhance the precision and reliability of the agreement estimates, providing visual and numerical insight into test interchangeability. MAE was used as a practical indicator of average estimation error. Together, these metrics allowed both statistical and clinical interpretation of test accuracy and utility.

## 3. Results

### 3.1. Participant Characteristics

Descriptive characteristics of the participants and VO_2_max outcomes are presented in Table 1. The athletes were, on average, approximately 64 years old, with a mean body mass index (BMI) reflecting an overweight profile despite regular competitive activity.

### 3.2. Comparison of VO_2_max Tests

**Figure 2:**
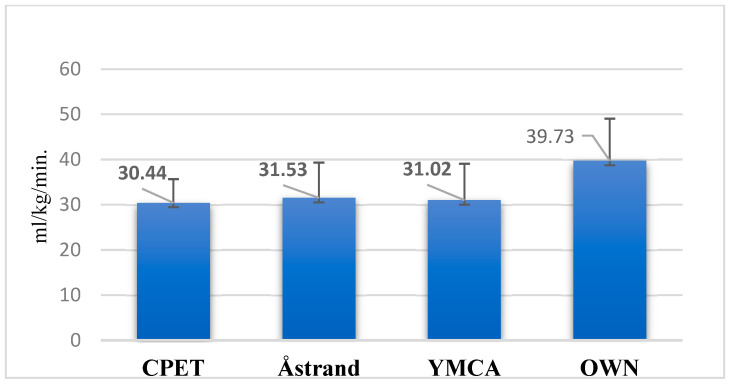
Mean maximal oxygen uptake (VO_2_max) values obtained from indirect tests compared with direct cardiopulmonary exercise testing (CPET). Note: Åstrand = Åstrand–Ryhming test; YMCA = YMCA test; OWN = Polar OwnIndex test. Test Accuracy: Bland–Altman Analysis.

Direct measurement via CPET in masters basketball players yielded a mean VO_2_max value used as the reference standard (see Table 1). Indirect tests generally produced higher VO_2_max estimates, with the Polar OwnIndex test showing the greatest deviation from the direct measurement, while the Åstrand–Ryhming test and YMCA protocols exhibited closer alignment. These observations indicate variability in predictive accuracy among the indirect methods, with Åstrand–Ryhming and YMCA tests demonstrating relatively smaller systematic biases (Figure 2).

**Figure 3:**
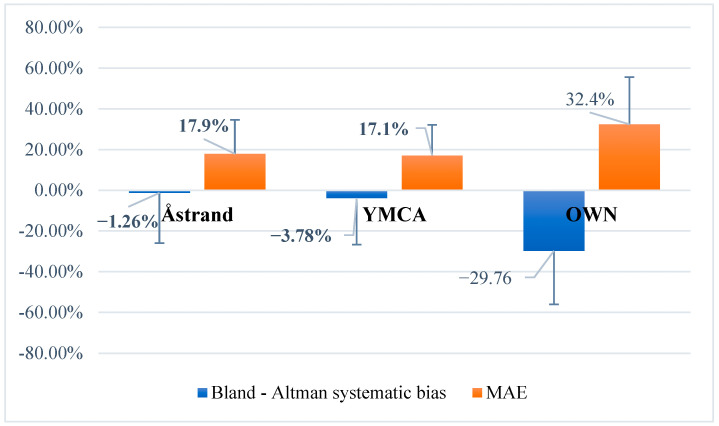
Bland–Altman analysis: systematic bias and mean absolute error (MAE, %) for each indirect test relative to cardiopulmonary exercise testing (CPET). Note: Åstrand = Åstrand–Ryhming test; YMCA = YMCA test; OWN = Polar OwnIndex test.

The Åstrand–Ryhming test and the YMCA test showed the lowest systematic bias and MAE values, whereas the Polar OwnIndex test exhibited a markedly higher bias and MAE (Figure 3). These findings indicate that the Åstrand–Ryhming and YMCA protocols demonstrate greater agreement with the direct CPET measurement compared with the Polar OwnIndex test.

### 3.3. Test Accuracy by Intensity

**Figure 4:**
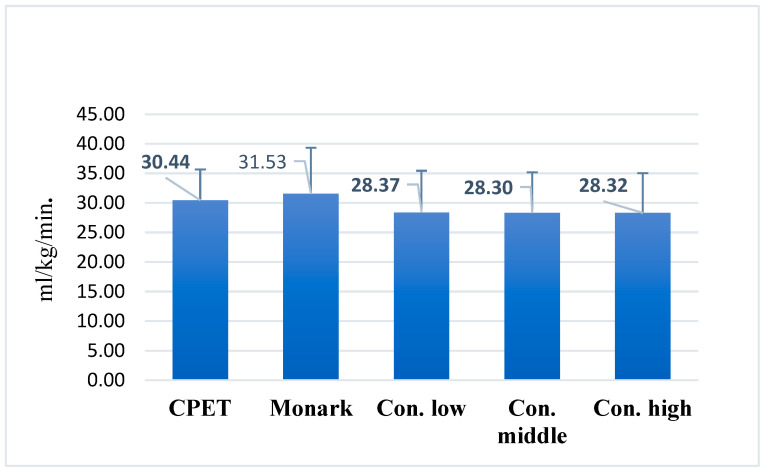
Mean maximal oxygen uptake (VO_2_max) values in the Åstrand–Ryhming test at different workload intensities. Note: Monark = Åstrand–Ryhming test on Monark ergometer; Con. low = low intensity on Concept2 BikeErg; Con. middle = moderate intensity; Con. high = high intensity; CPET = cardiopulmonary exercise testing.

In the Åstrand–Ryhming test conducted on the Concept2 BikeErg at varying intensity levels, mean VO_2_max values ranged from 28.30 ± 6.89 to 28.37 ± 7.09 mL/kg/min, showing minimal variation across low, moderate, and submaximal workloads (Figure 4).

**Figure 5:**
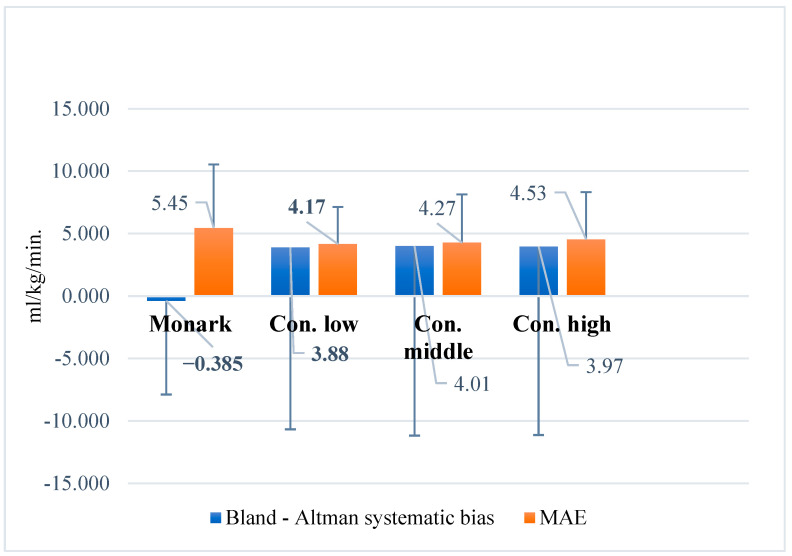
Bland–Altman plot of systematic bias and mean absolute error (MAE) in the Åstrand–Ryhming test according to workload intensity. Note: Monark = Åstrand–Ryhming test on Monark ergometer; Con. low = low intensity on Concept2 BikeErg; Con. middle = moderate intensity; Con. high = high intensity.

Systematic bias across the varying exercise intensities ranged from −0.385 to 4.01 mL/kg/min, whereas the MAE was consistently low, ranging from 4.17 to 4.53 mL/kg/min (Figure 5).

**Figure 6:**
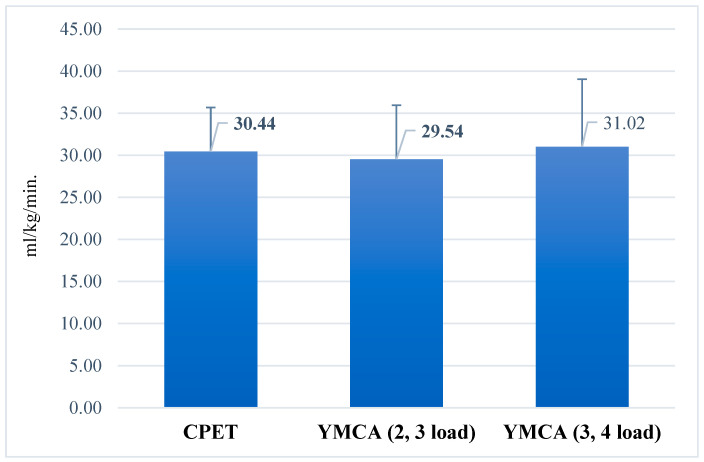
Mean maximal oxygen uptake (VO_2_max) values in the YMCA test at different workload intensities. Note: CPET = cardiopulmonary exercise testing; YMCA (2, 3 load) = YMCA test at low and moderate workload intensities; YMCA (3, 4 load) = YMCA test at moderate and high workload intensities.

Using the YMCA protocol, mean VO_2_max values varied according to workload levels, ranging from 29.54 ± 6.41 mL/kg/min at low and moderate workloads to 31.02 ± 8.04 mL/kg/min at moderate and submaximal workloads (Figure 6). This illustrates how different workload intensities influence the estimated VO_2_max.

**Figure 7:**
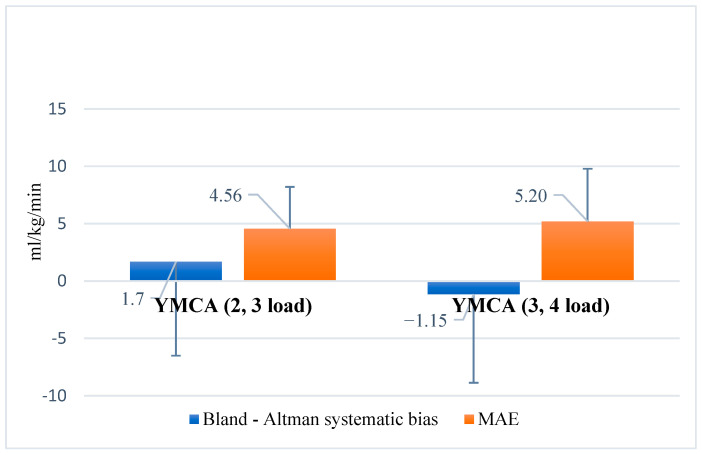
Bland–Altman plot of systematic bias and mean absolute error (MAE) in the YMCA test according to workload intensity. Note: YMCA (2, 3 load) = YMCA test at low and moderate workload intensities; YMCA (3, 4 load) = YMCA test at moderate and high workload intensities.

Bland–Altman analysis (Figure 7) revealed a small systematic bias, ranging from −1.15 to 1.7, with a MAE of approximately 5%. These results indicate good agreement between the YMCA-estimated VO_2_max and the reference CPET measurements, confirming that the protocol provides reasonably accurate estimations across varying workloads.

### 3.4. Correlation Analysis

Statistically significant positive correlations (*p* < 0.001) were found between all indirect test results and directly measured VO_2_max. The strongest correlation was observed in the Åstrand–Ryhming test at low workload (*r* = 0.749), followed by the YMCA protocol (*r* ≈ 0.52) and the Polar OwnIndex test (*r* = 0.517). Detailed results are presented in Table 2.

The results revealed notable differences between direct and indirect VO_2_max assessments. The Åstrand–Ryhming and YMCA protocols demonstrated the lowest error rates and strongest correlations with CPET, while the Polar OwnIndex test consistently overestimated VO_2_max.

## 4. Discussion

VO_2_max is a key marker of cardiorespiratory fitness, particularly in athletes. While CPET is the gold standard, indirect submaximal protocols are widely applied due to their accessibility and lower cost. However, their accuracy depends on the characteristics of the population assessed. Although VO_2_max estimation methods are well studied in general and athletic groups, research on masters basketball players is scarce, with most studies focusing on younger or professional athletes. This gap limits understanding of the validity of indirect VO_2_max tests in aging athletes, whose physiological adaptations differ from younger cohorts. Since heart rate variability is greater at lower exercise intensities [17], and resting-state algorithms such as the Polar OwnIndex test may not reflect sport-specific adaptations in older athletes, it was hypothesized that the Åstrand–Ryhming and YMCA protocols would show better agreement and lower bias relative to CPET. This study is the first to compare direct and indirect VO_2_max assessments in masters basketball players, providing new insights into test validity in this unique athletic population.

In this study, three indirect VO_2_max tests were compared with CPET results in experienced masters basketball players. The results confirmed the hypothesis: the Åstrand–Ryhming and YMCA cycle ergometer tests produced relatively accurate VO_2_max estimates, with low systematic errors (ranging from −0.385 to +4.01 mL/kg/min for Åstrand–Ryhming and from −1.15 to +1.7 mL/kg/min for YMCA) and low MAE values (ranging from 4.17 to 4.53 mL/kg/min for Åstrand–Ryhming). In contrast, the Polar OwnIndex test demonstrated substantially lower accuracy, with a large systematic bias of −9.29 mL/kg/min (representing a 29.76% systematic error) and an absolute error of approximately 9.85 mL/kg/min (MAE: 32.4%).

These findings align with previous literature [27], which indicates high individual prediction error for the Åstrand–Ryhming test, especially in men, yet still supports its reliability at the group level. The Ekblom-Bak test, not included in this study, is considered in the literature to offer even greater accuracy due to its reliance on heart rate response between two submaximal workloads [28]. A comparative study found that VO_2_max values obtained from submaximal tests, such as YMCA and Åstrand–Ryhming protocols, were significantly lower than those measured by CPET. While these methods may yield acceptable accuracy at the population level, they lack sufficient precision for assessing individual physiological capacity [29]. The observed acceptable group-level accuracy of our submaximal protocols (Åstrand–Ryhming and YMCA) aligns with broader methodological discussions concerning the utility of submaximal CPET approaches in specific populations. For instance, Wiecha et al. [23] and others emphasize that controlled submaximal assessments offer a practical balance between accessibility and the need for physiological rigor. While our cycle ergometer protocols use extrapolation from fixed submaximal workloads, their demonstrated capacity to reliably estimate VO2max highlights their practical value when direct maximal CPET is impractical.

The average VO_2_max in our cohort of masters basketball players measured via CPET was 30.44 mL/kg/min, which is lower than in younger basketball athletes, especially guards, who typically report higher VO_2_max values due to greater training intensity and frequency [30].

This overestimation in the Polar OwnIndex test may be attributed to the algorithm’s reliance on resting heart rate and demographic variables, which do not adequately reflect the cardiovascular efficiency or training adaptations present in older, physically active individuals. In masters athletes, lower resting heart rates and altered autonomic regulation may lead to inflated VO_2_max predictions when using static models. Furthermore, the algorithm does not account for sport-specific conditioning or individual variability in stroke volume and oxygen extraction, which are critical determinants of aerobic capacity. A further limitation specific to the Polar OwnIndex test method is that its predictive algorithms are embedded in proprietary software and firmware. Consequently, our findings are strictly applicable to the investigated version of the equipment, as test outcomes may change with subsequent updates.

YMCA testing protocols have been widely used in various populations. However, studies comparing them to CPET have reported varying levels of accuracy, possibly due to differences in test implementation and the modality used for comparison, such as treadmill versus cycle ergometer [31,32]. A large-scale study involving over 263,000 participants analyzed the accuracy of the Åstrand–Ryhming test, revealing high individual error due to physiological and psychological factors such as stress, temperature, and anxiety. The mean deviation was about 0.07 l/min, with coefficients of variation reaching 18% in men and 13.1% in women [27].

In this study, strong correlations with CPET results were found for the Åstrand–Ryhming test at low workloads (*r* = 0.749) and moderate workloads (*r* = 0.636). Moderate correlations were observed for the highest Åstrand–Ryhming workload (*r* = 0.517), YMCA tests at all intensities (*r* ≈ 0.52), and Polar OwnIndex test results (*r* = 0.517). Our findings revealed significant differences between indirect VO_2_max estimation methods when compared to the CPET reference standard. Similarly to findings by Santtila et al. (2013), who reported relative errors of 0.9–2.7% and correlations of *r* = 0.80–0.84 for the MILFIT cycling test, several protocols in our study demonstrated acceptable group-level accuracy, but varied in their capacity to detect individual differences [33].

The YMCA test (stages 3 and 4) showed the smallest systematic bias (from −1.15 to 1.7) and the most accurate group-level VO_2_max estimate (31.02 ± 8.04 mL/kg/min), closely matching the CPET result (30.44 ± 5.23 mL/kg/min). However, the Åstrand–Ryhming test, particularly the low-intensity BikeErg protocol, yielded the highest correlation with CPET (*r* = 0.749), indicating better sensitivity in detecting relative differences between individuals. This trend aligns with Beltz et al. (2016) [34], who emphasized the importance of evaluating both absolute accuracy and the ability to capture inter-individual physiological variation.

In line with previous findings [23], the Polar OwnIndex test demonstrated the largest systematic (29.76%) and absolute (MAE: 32.4%) errors, further confirming its limited validity in older athletic populations. These findings provide critical evidence for selecting appropriate VO_2_max estimation tools tailored to the needs of masters athletes.

Strengths and Limitations of the Study. The main strength of this study is its focus on a relatively under-researched but practically important population—physically active masters basketball players. By addressing this specific demographic, the study expands the existing body of knowledge with new empirical data. Moreover, the use of a direct and highly accurate method for assessing VO_2_max—CPET with breath-by-breath gas analysis—provided a reliable reference standard for evaluating the validity of indirect methods.

By comparing several indirect VO_2_max estimation protocols with different formats and intensity levels, the study was able to assess not only the test types themselves but also how protocol parameters influence measurement accuracy. The application of robust analytical techniques, including Pearson correlations and Bland–Altman analysis, added methodological rigor and enabled nuanced comparison across tests.

Another significant strength lies in the relative homogeneity of the participant sample—physically active male masters basketball players—which helped reduce confounding variability and increased the internal validity of the findings. At the same time, the homogeneous sample (composed exclusively of male masters basketball players) imposes significant limitations in terms of external validity. The findings may not be directly generalizable to female masters athletes or to individuals engaged in distinct sports (e.g., endurance or intermittent sports other than basketball), nor to less active populations. While test–retest reliability was not formally assessed, all testing sessions were conducted under standardized and controlled conditions, minimizing random variance.

Additionally, the study did not directly measure psychological factors such as motivation, anxiety, or fatigue, all of which may influence submaximal test outcomes. Future studies should include psychometric assessments to improve interpretation and better account for individual variability.

Implications for Practice. These findings have practical implications for sports professionals, coaches, and healthcare practitioners working with masters athletes and older active adults. We recommend that the Åstrand–Ryhming test performed at low or moderate workloads be adopted as the primary, practical and reasonably accurate alternative for estimating VO_2_max when direct CPET is unavailable. The YMCA protocol, although slightly less accurate, remains a viable screening or educational tool. In contrast, the Polar OwnIndex test must be interpreted with high caution due to its tendency to systematically overestimate aerobic capacity (by nearly 30%). These results provide a clear, evidence-based hierarchy for selecting appropriate fitness assessment tools in both training and clinical contexts, balancing precision, accessibility, and time-efficiency.

Recommendations for Future Research. The most crucial future research is to determine if these indirect protocols can track changes in fitness status over time. Longitudinal studies are needed to evaluate the sensitivity of the Åstrand–Ryhming and YMCA tests in reflecting true physiological improvements or declines measured by CPET. Furthermore, future studies should expand the participant pool to include diverse populations across gender, age, and fitness levels to improve the generalizability of findings. It would also be valuable to investigate the validity of these testing protocols in other sports and lifestyle groups. Moreover, emerging protocols such as the Ekblom–Bak test—which has shown promising correlations with direct VO_2_max in other studies—warrant validation in older athletic populations. Future research should also address test–retest reliability and incorporate psychological measures (e.g., motivation, perceived exertion, anxiety) to better understand how psychological variables influence test performance and outcome validity.

## 5. Conclusions

Compared to direct CPET, the Åstrand–Ryhming and YMCA tests demonstrated the lowest systematic and absolute errors, confirming their suitability for practical and reliable VO_2_max assessment in masters athletes. In contrast, the Polar OwnIndex test consistently overestimated VO_2_max and should be applied cautiously in this population. These results fill a crucial gap in validation studies focused on older, physically active athletes, providing evidence-based guidance for test selection. By adopting the Åstrand–Ryhming or YMCA protocols, sports medicine practitioners and trainers working with masters athletes can implement more accurate, cost-effective, and accessible fitness assessments, ultimately enhancing training personalization and monitoring in this growing athletic demographic.

## Figures and Tables

**Table 1:** Descriptive statistics of participants and VO_2_max assessments.

Variable	Mean ± SD
Age (years)	64.28 ± 7.90
Height (cm)	182.43 ± 6.69
Body mass (kg)	90.97 ± 14.76
BMI (kg/m^2^)	27.24 ± 3.63
CPET VO_2_max (ml/kg/min)	30.44 ± 5.23
Åstrand–Ryhming test (Monark) VO_2_max (mL/kg/min)	31.53 ± 7.80
Polar OwnIndex test VO_2_max (mL/kg/min)	39.73 ± 9.30
YMCA (workloads 3 & 4) VO_2_max (mL/kg/min)	31.02 ± 8.04

Note: BMI = body mass index; CPET = cardiopulmonary exercise testing; VO_2_max = maximal oxygen uptake; SD = standard deviation.

**Table 2:** Pearson correlation coefficients between indirect VO_2_max test results and direct CPET measurement.

Test	Pearson’s *r* (Correlation with CPET VO_2_max)	*p*-Value
Polar OwnIndex test	*r* = 0.517	*p* < 0.001
Åstrand–Ryhming test (Monark)	*r* = 0.403	*p* = 0.016
Åstrand–Ryhming test (low intensity)	*r* = 0.749	*p* < 0.001
Åstrand–Ryhming tests (moderate intensity)	*r* = 0.636	*p* < 0.001
Åstrand–Ryhming tests (high intensity)	*r* = 0.594	*p* < 0.001
YMCA (workloads 2 & 3)	*r* = 0.519	*p* < 0.001
YMCA (workloads 3 & 4)	*r* = 0.520	*p* < 0.001

## Data Availability

The datasets presented in this article are not readily available because the study is part of an ongoing research project and the data have not yet been deposited in a public repository. The data will be made available after the project concludes on 21 December 2026, in accordance with institutional and funding regulations. Requests to access the data prior to that date should be directed to the corresponding author.
